# Roles of myokines in osteoporosis under physiological and diabetic conditions

**DOI:** 10.3389/fendo.2025.1600218

**Published:** 2025-06-11

**Authors:** Xing Ji, Xinhua Hu, Taotao Xu, Wanlei Yang

**Affiliations:** ^1^ Department of Pharmacology, School of Medicine, Hangzhou City University, Hangzhou, China; ^2^ Department of Pharmacology, Zhejiang University School of Medicine, Hangzhou, China; ^3^ Center of Clinical Pharmacology, Second Affiliated Hospital, School of Medicine, Zhejiang University, Hangzhou, Zhejiang, China; ^4^ Department of Orthopedics Surgery, The First Affiliated Hospital of Zhejiang Chinese Medical University (Zhejiang Provincial Hospital of Chinese Medicine), Hangzhou, China

**Keywords:** myokines, diabetes, bone metabolism, osteoporosis, osteoblast lineage cells, osteoclast*, T1DM, T2DM

## Abstract

The musculoskeletal system is not only closely linked anatomically, but muscle-derived myokines also play a crucial role in bone development and metabolism beyond the effects of mechanical force. Myokines are essential in muscle-bone crosstalk, significantly influencing bone remodeling and metabolism. In the context of diabetes, including both type 1 (T1DM) and type 2 (T2DM), changes in myokine expression have a substantial impact on bone metabolism, leading to an increased risk of osteoporosis. This review provides a comprehensive examination of the roles of key myokines in regulating osteoblast lineage cells and osteoclast activity. We highlight how different myokines can either promote or inhibit bone formation and resorption and discuss their altered expression levels under diabetic conditions. A deeper understanding of the multifaceted roles of myokines may open new avenues for treating osteoporosis, particularly in diabetic patients.

## 1 Introduction

Myokines, a term first introduced in 2003 by Pedersen et al. (1), are cytokines and other peptides produced and released by muscle fibers in response to muscular contractions. These molecules act in an autocrine, paracrine, or endocrine manner, influencing not only muscle function but also other tissues, including bone (2, 3). Myokines play crucial roles in muscle hypertrophy, metabolism, and inflammation regulation (4, 5), and they are increasingly recognized for their role in muscle-bone crosstalk, particularly in the context of diabetes (6).

Osteoporosis is a prevalent and serious condition characterized by decreased bone mass and the structural deterioration of bone tissue, which leads to an increased risk of fractures (7). Recent studies increasingly demonstrate the significant impact of factors secreted by other organs on bone health (8, 9). Our review specifically focuses on the role of muscles, which are anatomically closest to bone, beyond the traditionally acknowledged mechanical effects. Myokines play a central role in regulating osteoblast (OB) and osteoclast (OC) activities, thereby contributing to the development and progression of osteoporosis. In particular, myokines such as irisin, myostatin, and interleukin-6 (IL-6) have been found to influence bone metabolism by modulating the differentiation and functional activities of bone-forming osteoblasts and bone-resorbing osteoclasts.

Diabetes is a chronic metabolic disease characterized by persistent hyperglycemia, resulting from defective insulin secretion or action, leading to impaired glucose, lipid, and protein metabolism. Beyond its impact on glycemic control, diabetes negatively affects bone health, increasing the risk of osteoporosis and fractures due to changes in bone metabolism and microarchitecture (10, 11). Growing evidence highlights a significantly elevated fracture risk in people with diabetes, particularly among those with T1DM (12). In a recent meta-analysis, individuals with type 1 diabetes had a significantly increased risk of both hip and non-vertebral fractures. The relative risk of hip fracture was 4.93 (95% CI: 3.06–7.95), while the risk for non-vertebral fractures was 1.92 (95% CI: 0.92–3.99). Notably, hip fracture risk was more pronounced in younger individuals (13). In T2DM, the increase in fracture risk is more modest but becomes substantial in individuals with low BMI, prolonged disease duration, or insulin therapy, where the risk may rise by more than 20% (14, 15). These findings underscore the importance of individualized fracture risk assessment in the context of diabetes. Notably, myokines are key factors that influence systemic metabolism, prompting us to explore how these myokines change within the context of diabetes and their effects on bone.

We have comprehensively cataloged all known myokines and detailed their specific effects on osteoblasts lineage cells and osteoclasts. Special emphasis is placed on the circulating concentrations of these myokines and their alterations in the context of diabetes. Additionally, we critically examine the impact of these changes on bone metabolism in the context of diabetes, highlighting how variations in myokine levels influence bone formation, resorption, and overall skeletal integrity in diabetes. This review aims to provide a comprehensive and systematic review of the role of myokines in bone metabolism, with a particular focus on their role in diabetic conditions. Through this study, we hope to provide a theoretical basis and identify novel therapeutic targets for the prevention and treatment of osteoporosis associated with diabetes.

## 2 The musculoskeletal unit in diabetes: traditional and emerging views

### 2.1 Traditional view of mechanical coupling between muscles and bones

The interaction between bones and skeletal muscles has traditionally been regarded as a mechanically driven relationship, where muscles generate forces that stimulate bone adaptation, while bones provide structural support and serve as attachment sites for muscles (16, 17). Mechanical loading is a key determinant of bone strength, influencing osteoblast activity, bone mineralization, and trabecular architecture. During musculoskeletal development, these mechanical forces ensure proper cell fate determination, structural organization, and functional integration of bones, joints, tendons, and muscles. Hippo signaling (YAP1), Indian hedgehog (IHH), and parathyroid hormone-related protein (PTHrP) are among the key regulators of this process (18). However, in diabetes, the mechanical loading response of bone is impaired due to muscle wasting, neuropathy, and altered mechanotransduction signaling, leading to accelerated bone loss (19). While mechanical forces remain essential for bone adaptation, emerging evidence highlights the role of myokines—muscle-derived cytokines that influence bone metabolism through endocrine signaling.

### 2.2 Myokines: a new frontier in the treatment of diabetic osteoporosis

Myokines act as key mediators in the muscle-bone crosstalk, regulating the balance between bone formation and resorption. Their secretion is modulated by physical activity, metabolic status, and inflammatory signals, and they are significantly altered in diabetes and its complications. In both T1DM and T2DM, dysregulated myokines affect osteoblasts, osteoclasts, and mesenchymal stem cells (MSCs) and disrupt the balance between bone formation and resorption. These changes contribute to diabetes-induced osteoporosis, leading to impaired bone remodeling, increased fragility, and a higher risk of fractures (20). The mechanisms through which specific myokines influence bone metabolism in the context of diabetes will be discussed in detail in the following sections. Understanding the role of myokines within the musculoskeletal unit offers a novel approach to managing bone complications in the context of diabetes, providing insights into potential targeted therapies that leverage muscle-derived factors to enhance both muscle function and bone health in people with diabetes.

## 3 Myokines and bone metabolism

### 3.1 Overview of myokines and their impact on bone resorption and formation

**Figure 1:**
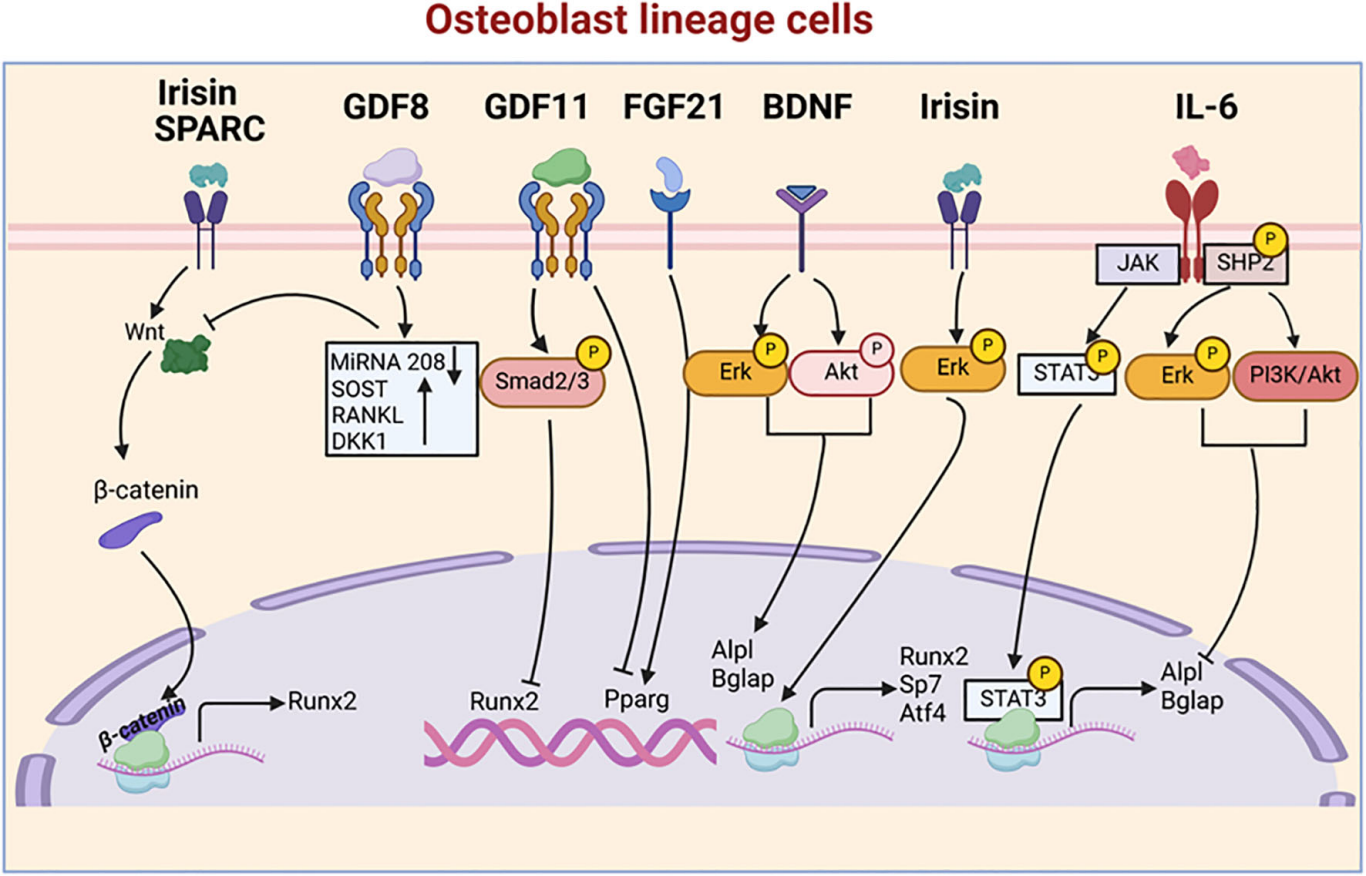
Myokine signaling pathways regulating osteoblastogenesis. This figure illustrates the complex network of myokine signaling pathways that control osteoblast differentiation and function. Key myokines, including Irisin, GDF8, GDF11, FGF21, BDNF, Sparc, and IL-6, activate various signaling cascades, such as Smad, Erk, Akt, and STAT3 pathways. These pathways converge on key transcription factors, including Runx2, Pparg, and Sp7, which regulate the expression of osteoblast-specific genes like Alpl and Bglap.

**Figure 2:**
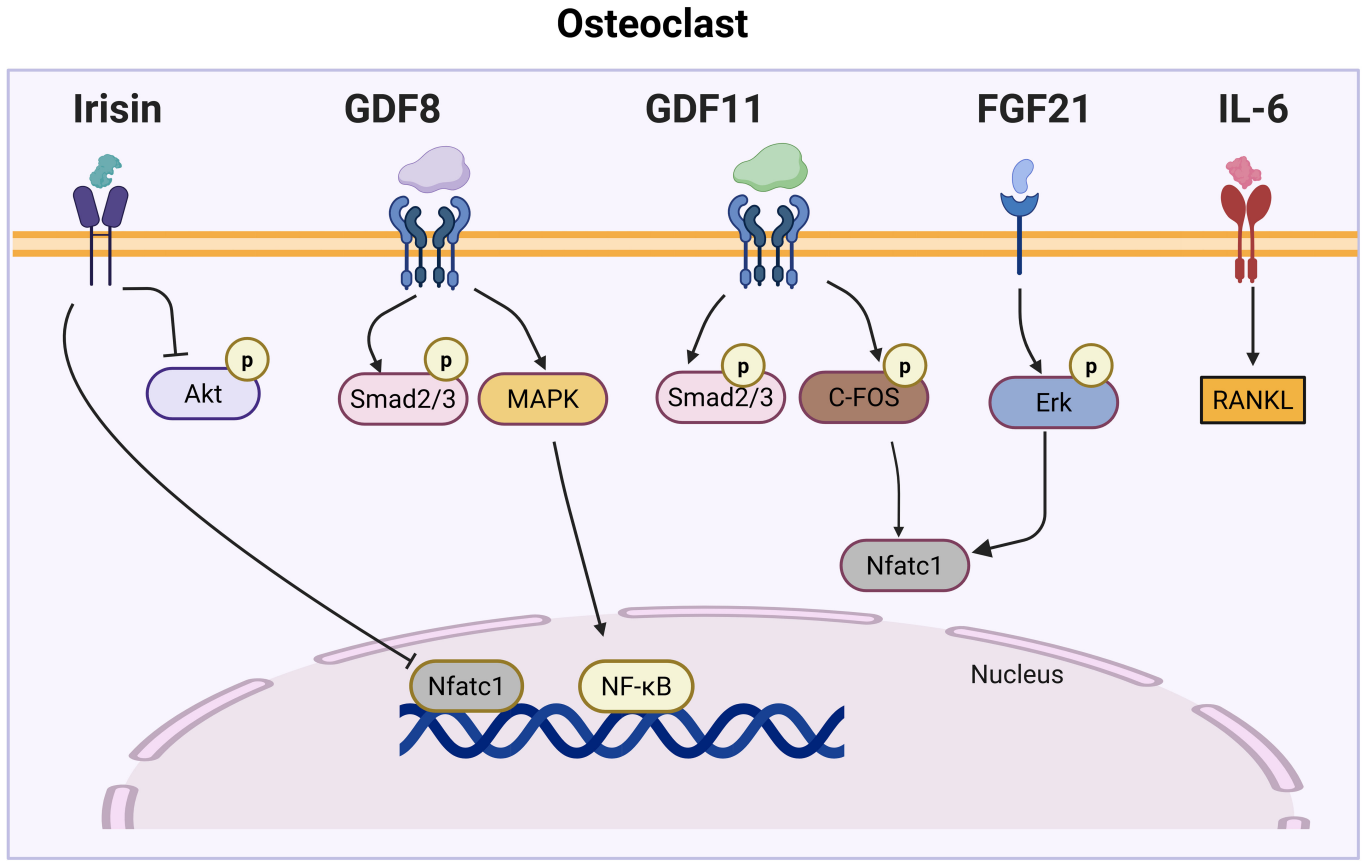
Myokine signaling pathways regulating osteoclast differentiation. This figure illustrates the complex network of myokine signaling pathways that control osteoclast differentiation. Key myokines, including Irisin, GDF8, GDF11, FGF21, and IL-6, activate various signaling cascades, such as Akt, MAPK, Smad2/3, and Erk pathways. These pathways converge on key transcription factors, such as NFATc1 and NF-κB, which regulate the expression of osteoclast-specific genes.

Myokines are muscle-derived signaling molecules that influence bone metabolism through a diverse range of mechanisms, impacting both OB and OC. This section explores the phenotypes observed in mouse models for each myokine, further enhancing our understanding of their impact on both OB and OC (**Table 1**). Additionally, we summarize their roles in bone formation and resorption, while also examining the molecular pathways they regulate (**Figures 1**, **2**), highlighting their contributions to skeletal homeostasis and potential implications for bone health and disease. Their effects are highly context-dependent, influenced by factors such as concentration, receptor availability, and intracellular signaling crosstalk.

**Table 1:** Summary of mouse models evaluating the impact of various myokines on bone mass and skeletal phenotype.

Myokines	Stains	Sex	Age	Bone Phenotype	Name, Year [Ref.]
The Impact of Myokines on Bone: A Summary of Mouse Models					
**Irisin**	FNDC5 KO	female	5 M	No bone phenotype	Kim, 2018 [21]
	MCK-FNDC5 Tg	–	2 M	Decrease in bone mass	Estell, 2020 [22]
**GDF8**	GDF8 KO	male and female	9-10 M	Increase in bone mass	Hamrick, 2002 [23]
**GDF11**	GDF11 KO	–	E11.5	various skeletal abnormalities	McPherron, 1999 [24]
**IL-6**	IL-6 KO	female	4 M	No trabecular bone loss, cortical bone volume was significantly lower	Poli, 1994 [25]
	IL-6 KO	male	20-week-old	No bone phenotype	Li, 2021 [26]
**BDNF**	central BDNF depletion,Bdnf^2lox/2lox^/93	male and female	3 and 6 M	Increase in bone mass	Camerino, 2012 [27]
**METRNL**	Metrnl KO	male and female	6-week-old	No bone phenotype	Huang, 2022 [28]
**FGF21**	FGF21 KO	female	16-week-old	Increase in bone mass	Bornstein, 2014 [29]
	FGF21 KO	male	4 M	Increase in bone mass	Wei, 2014 [83]
	FGF21-Tg Mice	male	6 M	Decrease in bone mass	
	FGF21 KO	male	15-week-old	No bone phenotype	Li, 2017 [31]
**Musclin**	Ostn^LacZ/LacZ^	male	8-week-old	Decrease in bone mass	Watanabe-Takano, 2021 [32]
	SAP-Ostn-Tg	male and female	10-week-old	No bone mass phenotype, but longer bone	Kanai, 2017 [33]
**SPARC**	Osteonectin-deficient	male and female	11,17 and 36-week-old	severe trabecular osteopenia	Delany, 2000 [34]
**DKK3**	Dkk3 KO	male and female	P0-12 M	No bone phenotype	Barrantes Idel, 2006 [35]

This table includes genetically modified strains for key myokines such as Irisin, GDF8, GDF11, IL-6, BDNF, METRNL, FGF21, Musclin, SPARC, and DKK3. Bone phenotypes were assessed across sexes and age ranges as reported in the corresponding studies.

### 3.2 Specific myokines and their effects on bone cells

#### 3.2.1 Irisin

Irisin, a myokine released during exercise, plays a crucial role in energy metabolism by promoting the conversion of white adipose tissue (WAT) into thermogenic brown fat (BAT)-like cells, enhancing energy expenditure and glucose homeostasis (36, 37). It binds to αV integrins, particularly αV/β5, initiating signaling pathways that influence bone and fat metabolism (21). In bone metabolism, irisin exhibits tissue-specific effects. Irisin is produced by the cleavage of fibronectin type III domain-containing protein 5 (FNDC5), and it is released into the bloodstream. While global FNDC5 knockout (KO) does not significantly affect bone mass, it confers resistance to ovariectomy (OVX)-induced bone loss (21). In contrast, muscle-specific FNDC5 KO reduces bone mass, indicating a regulatory role in skeletal health (22). Intermittent low-dose irisin injections enhance cortical bone mineral density (37), likely through osteoblast stimulation via the Wnt/β-catenin and MAPK pathways, promoting autophagy and osteogenic differentiation (38–40). Additionally, irisin activates AMP-activated protein kinase (AMPK), influencing inflammatory cytokines and promoting the M2 macrophage phenotype, which enhances osteogenesis (41). Irisin’s impact on osteoclasts is dose-dependent. At low concentrations (10 ng/mL), it promotes osteoclast differentiation (22), whereas higher doses (120–480 ng/mL) suppress RANKL-induced osteoclastogenesis by inhibiting Akt phosphorylation and NFATc1 activation (42). Variability in reported circulating irisin levels (3–5 ng/mL vs. commercial kit measurements of ~100 ng/mL) underscores the importance of dosage and detection methods in interpreting its skeletal effects (43, 44). These findings suggest irisin’s role in bone metabolism is complex and context dependent.

#### 3.2.2 GDF8

Myostatin (GDF8), a TGF-β superfamily member, is primarily expressed in skeletal muscle as a negative regulator of muscle growth (45), but it also plays a key role in bone metabolism. It circulates in an inactive form, bound to a propeptide, and is activated by BMP1 or Tolloid metalloproteinases. GDF8 is inhibited by follistatin and FLRG (Follistatin-Related Gene) and signals through the ActRIIB receptor, triggering TGF-β signaling that regulates bone cell activity (46). GDF8 is critically involved in regulating the proliferation and differentiation of mesenchymal stem cells (47, 48). Studies in GDF8 KO mice have demonstrated significant reductions in body fat and notable increases in bone mineral density and strength (23), observed in the appendicular skeleton, vertebral column, and mandible (49, 50). In osteoblasts and osteocytes, GDF8 upregulates SOST, DKK1, and RANKL, while suppressing miR-218, thereby inhibiting osteoblast differentiation (51). It also enhances the mechanosensitivity of bone marrow stromal cells, promoting an osteogenic response to mechanical loading (52, 53). However, GDF8 inhibition alone has limited effects on bone formation, particularly in aged mice, and is less effective than ActRIIB-Fc, which blocks multiple ligands, including BMPs (54). In osteoclasts, GDF8 enhances RANKL-mediated osteoclastogenesis via the Smad2-NFATC1 axis and promotes differentiation through Ccdc50-regulated NF-κB and MAPK signaling (55, 56). Although GDF8-modified osteocytic exosomes can be internalized by osteoclasts, they have minimal impact on osteoclast differentiation, underscoring the multifaceted nature of GDF8’s role in bone metabolism (51).

#### 3.2.3 GDF11

Similar to GDF8, GDF11, another TGF-β superfamily member (57, 58), plays a significant role in skeletal development and adult bone homeostasis. Homozygous Gdf11-null mice (Gdf11^-/-^) exhibit severe skeletal abnormalities, including anterior homeotic transformations and tail absence, while heterozygous (Gdf11^+/-^) mice display milder defects (24). In adult mice, daily administration of recombinant GDF11 (rGDF11) reduces trabecular bone volume, increases osteoclast number, and decreases osteoblast activity, leading to lower mineral apposition and bone formation rates (59). At the cellular level, GDF11 inhibits osteoblast differentiation by activating Smad2/3 signaling, which suppresses Runx2 expression (60), thereby impairing osteoblastic differentiation of bone marrow stromal cells (BMSCs). While some studies suggest that GDF11 promotes osteoblastogenesis through PPARγ inhibition (61), others indicate that blocking GDF11 enhances bone formation, highlighting ongoing debate regarding its precise role. In osteoclasts, GDF11 promotes Smad2/3-mediated c-Fos upregulation, leading to increased Nfatc1 expression, which enhances osteoclast differentiation and activity (59). This osteoclastogenic effect aligns with the bone loss observed following rGDF11 administration.

#### 3.2.4 IL-6

Interleukin-6 (IL-6), a pleiotropic cytokine (62, 63), was initially identified in immune regulation but was later recognized as a myokine produced by muscle fibers during contraction (64). IL-6 signaling occurs through three pathways: classic signaling via membrane-bound IL-6R, trans-signaling through soluble IL-6R (sIL-6R), and trans-presentation, where dendritic cells present IL-6 to T cells via IL-6R (65). Studies on IL-6 KO and transgenic mice reveal its complex role in bone metabolism. Female IL-6 KO mice (4 months old) showed preserved trabecular bone despite ovariectomy but had reduced cortical bone volume (25), while male IL-6 KO mice (20 weeks old) showed no significant differences in bone mass (26). In contrast, IL-6 transgenic mice, with chronically elevated IL-6, exhibited smaller skeletons, reduced trabecular and cortical bone, and impaired ossification, likely due to osteoclast overactivity and osteoblast suppression (66). At the cellular level, IL-6 modulates osteoblast activity through different pathways. Since osteoblasts have low membrane-bound IL-6Rα, IL-6 primarily acts through trans-signaling, requiring sIL-6R. IL-6 suppresses osteoblast differentiation via SHP2/MEK2/ERK and SHP2/PI3K/Akt2 pathway s (67), while the gp130-STAT1/3 axis promotes bone formation. Disruption of gp130-STAT1/3 signaling (gp130ΔSTAT/ΔSTAT mice) leads to premature growth plate closure, reduced bone length, and impaired skeletal growth, underscoring its essential role in maintaining bone elongation (68). Conversely, IL-6 strongly promotes osteoclastogenesis by upregulating RANKL in osteoblasts and stromal cells, facilitating osteoclast differentiation. Muscle-derived IL-6, produced during exercise, is required for osteoblast-mediated osteoclast activation and the release of bioactive osteocalcin, which enhances exercise capacity (69). In gp130Y757F/Y757F mice, where the SHP2/Ras/MAPK pathway is impaired, increased osteoclastogenesis and bone resorption lead to osteopenia, highlighting the distinct role of this pathway in bone turnover regulation (68).

#### 3.2.5 Brain-derived neurotrophic factor

Brain-Derived Neurotrophic Factor (BDNF), traditionally known for its role in the central nervous system, has also been identified as a myokine secreted by skeletal muscle during contraction. However, studies suggest that muscle-derived BDNF does not enter systemic circulation, indicating a predominantly local effect within muscle tissue (70). Despite this, BDNF has been increasingly linked to bone development and remodeling. BDNF and its receptor TrkB are co-expressed in chondrocytes of the epiphyseal growth plate, active osteoblasts, and trabecular bone, suggesting a regulatory role in skeletal metabolism (71). Mice with central BDNF depletion exhibit increased bone mass, elongated femurs, and elevated bone mineral density (BMD) and bone mineral content (BMC), highlighting its systemic influence on bone physiology (27). At the cellular level, BDNF enhances osteoblast activity by upregulating osteocalcin, alkaline phosphatase (ALP), bone morphogenetic protein-2 (BMP-2), and osteopontin via TrkB-mediated ERK1/2 and AKT signaling pathways, promoting osteoblast differentiation and migration (72, 73). In osteoclasts, BDNF indirectly regulates osteoclastogenesis by modifying the RANKL/OPG ratio. It increases osteoprotegerin (OPG) expression, which inhibits RANKL-RANK binding, thereby reducing osteoclast differentiation and bone resorption (74). Currently, direct experimental evidence linking muscle-derived BDNF to bone metabolism is lacking. Given its restricted local effects in muscle, further research is needed to clarify its role as a myokine in bone physiology.

#### 3.2.6 Metrnl

Metrnl (Meteorin-like) is a hormone-like protein involved in inflammation, metabolism, and muscle repair, primarily expressed in adipose tissue and skeletal muscle, with detectable levels in bone. It is secreted post-exercise by muscle cells and upon cold exposure by adipose tissue, promoting energy expenditure and glucose homeostasis through beige fat thermogenesis and anti-inflammatory pathways (75). Despite its expression in perichondrium and primary ossification centers during skeletal development, Metrnl-null mice exhibit no significant differences in bone length, bone volume, trabecular thickness, or cortical thickness compared to wild-type mice. Additionally, Metrnl upregulation after bone injury does not affect bone deposition or fracture healing, suggesting that Metrnl is not essential for bone mass maintenance or normal fracture repair *in vivo* (28). At the cellular level, Metrnl suppresses osteoblast differentiation by inhibiting AP-1 activity, as shown in *in vitro* studies (76). However, its precise effects on osteoclasts remain unclear.

#### 3.2.7 L-BAIBA

The study identifies β-aminoisobutyric acid (BAIBA) as a small molecule myokine produced by muscle cells in response to exercise (77). A small molecule secreted by skeletal muscle during exercise, exerts a protective effect on osteocytes through the Mas-Related G-Protein Coupled Receptor D (MRGPRD) receptor. The binding of L-BAIBA to MRGPRD activates signaling pathways that prevent ROS-induced mitochondrial breakdown, thereby maintaining osteocyte viability. *In vivo* experiments demonstrated that L-BAIBA supplementation prevented bone loss in a murine hindlimb unloading model, which is typically used to simulate conditions of disuse osteoporosis (78). The combination of L-BAIBA and sub-optimal mechanical loading significantly increased bone formation markers compared to either treatment alone (79).

#### 3.2.8 FGF21

Fibroblast Growth Factor 21 (FGF21), primarily secreted by the liver, also functions as a myokine, with its expression in skeletal muscle regulated by the PI3K/Akt1 pathway (80). While FGF21 is well known for its role in glucose and lipid metabolism (81, 82), recent studies suggest conflicting effects on bone health. FGF21-deficient mice exhibit a high-bone-mass phenotype, with increased bone formation and reduced bone resorption, whereas transgenic FGF21-overexpressing mice display lower bone mass, reduced trabecular bone volume, and decreased bone thickness and number (83). FGF21 also plays a role in lactation-induced bone remodeling, promoting bone resorption, with knockout studies indicating that FGF21 deficiency preserves bone mass during lactation (29). However, some studies suggest that FGF21 does not significantly impact bone mass or turnover under normal conditions, leaving its precise role in bone metabolism unclear (31). At the cellular level, FGF21 suppresses osteoblast differentiation by upregulating PPARγ, shifting bone marrow stromal cell fate toward adipogenesis rather than osteogenesis, leading to a reduction in bone formation and an increase in marrow adipocytes (83). In osteoclasts, FGF21 indirectly promotes osteoclastogenesis by upregulating IGFBP1 (insulin-like growth factor-binding protein 1) in the liver, which increases circulating IGFBP1 levels. IGFBP1 binds to integrin β1 receptors on osteoclast precursors, activating ERK phosphorylation and NFATc1, thereby enhancing osteoclast differentiation and bone resorption (30). Despite accumulating evidence, the role of FGF21 as a myokine in bone metabolism remains underexplored, with conflicting findings across different studies. Strain, age, and diet variations may contribute to these discrepancies, but they do not fully explain the observed differences in bone phenotype, highlighting the need for further targeted research.

#### 3.2.8 Musclin

Musclin, also known as osteocrin, is a secreted peptide primarily expressed in skeletal muscle and bone (84). As a myokine, Musclin is upregulated in response to exercise, enhancing mitochondrial biogenesis and function in skeletal muscle (85). It also plays a role in insulin sensitivity, glucose metabolism, and fat oxidation. In bone, Musclin regulates skeletal growth and adaptation to mechanical loading. Osteocrin-overexpressing mice (SAP-Ostn-Tg) exhibit significant skeletal overgrowth with elongated limbs and thicker growth plates, while OSTN-deficient mice (OstnLacZ/LacZ) display reduced bone length and trabecular bone volume, indicating that osteocrin is essential for normal bone development and load-induced bone formation (33). In osteocytes, osteocrin is a target gene of Sp7 (Osterix) and is crucial for dendrite formation and osteocyte network integrity. Deleting Sp7 in osteoblasts results in defective osteocyte dendrites, increased cortical porosity, and higher osteocyte apoptosis rates. Overexpression of osteocrin rescues these defects, highlighting its role in maintaining osteocyte function (86). At the cellular level, osteocrin promotes bone elongation and thickness by preventing CNP clearance via NPR-C, thereby amplifying the CNP/GC-B/cGMP signaling pathway (32). Periosteal osteoblasts upregulate osteocrin expression in response to mechanical loading, reinforcing its role in bone adaptation to stress.

#### 3.2.9 SPARC

SPARC (Secreted Protein Acidic and Rich in Cysteine), also known as Osteonectin, is a matricellular glycoprotein that plays a crucial role in bone mineralization, extracellular matrix (ECM) regulation, and cellular processes such as proliferation, migration, and adhesion (87–89). Initially identified as a bone-specific protein, SPARC was later recognized as a myokine, secreted by muscle cells in response to exercise and mechanical stretching (90). While circulating SPARC is rapidly degraded, its degradation products may have biological significance, though direct evidence remains limited. In bone, SPARC interacts with integrin α5β1, activating Wnt/β-catenin signaling, which is central to skeletal homeostasis (91). SPARC-null mice exhibit severe trabecular osteopenia, diminished bone formation, and impaired bone turnover, which worsens with age, leading to reduced bone mass and compromised mechanical integrity (34). At the cellular level, SPARC deficiency reduces both osteoblast and osteoclast number and activity, disrupting normal bone turnover and remodeling. This is accompanied by increased collagen maturity and altered mineral crystallinity, resulting in weakened bone structure and impaired remodeling dynamics (92, 93). Furthermore, osteonectin-null mice exhibit an exaggerated osteoclastic response to parathyroid hormone (PTH) treatment, leading to increased bone resorption instead of the expected anabolic effect, indicating dysregulation of the bone formation-resorption balance (94). Genetic studies have identified a 3’ UTR SNP1599 variant that modulates osteonectin expression via miR-433 binding, influencing bone mass. The SNP1599G variant (haplotype A), more common in osteoporotic patients, introduces a miR-433 binding site, reducing SPARC expression and impairing bone formation. Conversely, the SNP1599C variant (haplotype B), associated with higher SPARC levels, correlates with improved bone health due to the absence of miR-433-mediated repression (95). Despite its established role in bone biology, there is no direct evidence regarding the impact of muscle-secreted SPARC on bone metabolism, highlighting a gap for future research.

#### 3.2.10 DKK3

Dickkopf-3 (DKK3) is a member of the Dickkopf family, which plays a distinct role in bone compared to DKK1, a critical player in the Wnt signaling pathway within bone (96). Moreover, unlike mice deficient in Dkk1 or Dkk2, those with a Dkk3 KO do not exhibit any bone-related phenotypes (35, 97, 98). Recent research has identified DKK3 as a myokine (99), elevated levels of DKK3 have been detected in the bloodstream following exercise in both mice and humans, with *in vitro* studies showing that cyclic stretching (mimicking muscle contractions) enhances its secretion​. Although the direct relationship between DKK3 and diabetes is not well-studied, the protein’s involvement in CKD and CVD, both of which are common diabetes complications, suggests that DKK3 could be an indirect marker for managing diabetes-related complications (100–102). Further research is required to fully understand its role in diabetes, but its potential as a biomarker for conditions closely linked to diabetes is noteworthy​.

## 4 Evidence from animal models and human studies in diabetes

### 4.1 Overview of myokine research in animal models of diabetes-related bone disorders

Animal models have been instrumental in elucidating the relationship between diabetes, myokines, and bone health. Studies utilizing streptozotocin (STZ)-induced diabetic mice and genetically modified db/db mice have demonstrated how diabetes disrupts myokine expression and its subsequent impact on bone metabolism. In these models, altered myokine profiles are also closely linked to insulin resistance, a key metabolic feature of diabetes. Several myokines, including Irisin (103, 104), GDF8 (105, 106), GDF 11 (107), IL-6 (108, 109), Metrnl (110), and FGF21, influence insulin sensitivity by modulating key components of the insulin signaling pathway such as the insulin receptor, PI3K/Akt, and GLUT4. Despite extensive research on myokine-mediated modulation of insulin signaling in metabolic tissues, their interplay within bone cells remains insufficiently investigated.

### 4.2 Irisin and diabetic bone loss

Irisin has protective effects on bone health in diabetic models. In T2D mice, irisin prevents bone loss by attenuating inflammasome-associated pyroptosis signaling via the miR-150-FNDC5/Irisin/pyroptosis axis, making it a promising therapeutic target for osteoporosis associated with diabetes (111). Additionally, in T1D models, irisin reduces oxidative stress and prevents periodontal bone destruction, highlighting its broader role in bone protection under hyperglycemic conditions (112).

### 4.3 GDF8 and bone atrophy in diabetes

GDF8 acts as a negative regulator of muscle and bone mass. In T1D rats, weight-bearing treadmill running counteracts bone atrophy by downregulating GDF8 expression through the Activin A Receptor Type 2B (ActRIIB)/Smad2/3 pathway (113). Additionally, in T2D individuals, increased GDF8 expression in bone tissue has been linked to impaired fracture healing. Blocking myostatin with Follistatin (a natural MSTN inhibitor) enhances bone regeneration, suggesting its potential as a therapeutic approach for improving diabetic bone healing (114). Moreover, treating mice with soluble ActRIIB-Fc, an inhibitor of the activin/myostatin pathway, significantly improves bone mass, strength, and muscle function, underscoring the interplay between muscle-bone crosstalk in diabetic bone loss (115).

### 4.4 FGF21 and metabolic-bone homeostasis in diabetes

FGF21, a metabolic regulator, plays a complex role in diabetic bone metabolism. In diet-induced obese (DIO) mice, treatment with recombinant human FGF21 (rhFGF21) improved metabolic parameters—including body weight, glucose, insulin, and lipid levels—but did not cause significant bone loss. Parameters such as bone mineral density (BMD), trabecular thickness, and bone volume fraction (BV/TV) remained unchanged, suggesting that FGF21’s metabolic benefits do not necessarily compromise skeletal integrity in diabetes. However, the long-term effects of FGF21 on bone remodeling under chronic hyperglycemia remain unclear (31).

Importantly, insulin is a key regulator of FGF21 expression in skeletal muscle. By activating the PI3K/Akt1 signaling pathway, insulin induces upregulation of FGF21 mRNA and protein levels, as demonstrated in both *in vitro* and *in vivo* studies (80, 116). This represents a bidirectional regulatory mechanism, in which insulin not only modulates myokine production but may also amplify the downstream skeletal effects of specific myokines. Given that insulin promotes FGF21 expression in skeletal muscle, it is plausible that the insulin–FGF21 axis contributes to the regulation of bone metabolism, particularly under diabetic conditions.

Research indicates that the expression levels of certain myokines, including irisin and myostain, are significantly altered in these diabetic models. These alterations are associated with changes in bone density and microarchitecture, suggesting that myokines play a contributory role in diabetes-induced bone disorders. Notably, these findings highlight the potential of myokines as therapeutic targets to mitigate bone loss in the context of diabetes. However, translating these animal model findings to human clinical scenarios requires careful consideration, given the differences in myokine expression patterns and bone metabolism between species.

#### 4.4.2 Examination of human studies on myokines, bone density, and fracture

##### 4.4.2.1 Risk in diabetic patients

Clinical studies provide substantial evidence that myokines play a crucial role in bone metabolism in people with diabetes. Significant differences in circulating myokine levels have been observed between individuals with diabetes and non-diabetic controls, as summarized in **Table 2**. Among these, irisin levels exhibit opposite trends in different forms of diabetes. Patients with T1D show elevated irisin levels (117), whereas those with T2D have significantly reduced levels compared to healthy controls (118, 119). Similarly, GDF8 concentrations in healthy individuals typically range from 1 to 8 ng/mL (120, 121), but in T1D patients, GDF8 levels are significantly elevated, particularly in women, suggesting a stronger inhibitory effect on muscle and bone mass under insulin-deficient conditions (122). In contrast, T2D patients exhibit only a slight elevation in circulating GDF8, yet muscle GDF8 mRNA expression is upregulated (1.4-fold higher), indicating potential dysregulation in skeletal muscle metabolism (123). Serum GDF11 levels average 5.92 ± 2.52 ng/mL and exhibit a negative correlation with bone mineral density (BMD) at the lumbar spine, total hip, and femoral neck (124). Although GDF11 declines with aging, its levels do not appear significantly influenced by obesity or glycemic status in T2D (125). IL-6 plays a complex role in both T1D and T2D pathogenesis, with its concentrations in healthy individuals averaging 5.186 pg/mL but increasing substantially during acute inflammation and muscle activity (126). In T1D, IL-6 levels are elevated, contributing to autoimmune-mediated β-cell destruction (127). In T2D, IL-6 acts as a pro-inflammatory mediator linking adipose tissue inflammation to systemic insulin resistance (128). Elevated IL-6 levels have also been implicated in diabetes-induced bone loss, potentially through increased osteoclastogenesis and altered osteoblast function. Similarly, BDNF concentrations in healthy individuals typically range 32.69 ± 8.33 ng/mL (129), but both T1D and T2D patients exhibit lower circulating BDNF levels, suggesting that BDNF deficiency may contribute to impaired bone remodeling and neuro-metabolic dysfunction in diabetes (130, 131).

In healthy individuals, median circulating FGF21 levels generally fall between 0.1 and 0.2 ng/mL, although significant inter-individual variability exists, with concentrations ranging from 0.021 to 5.3 ng/mL. Serum FGF21 levels are significantly lower in T1DM patients compared to non-diabetic individuals (132), while in T2DM, circulating FGF21 levels are consistently elevated (133, 134). A deficiency in FGF21 disrupts the normal regulatory feedback mechanisms in adipose tissue, leading to impaired systemic lipid and glucose homeostasis (82). In control groups, musclin serum concentrations typically range from 66.35 to 104.33 ng/mL. Elevated musclin levels have been reported in individuals with T2DM (135, 136). Recent research has also suggested that SPARC plays a role in metabolic diseases such as obesity and T2DM, where it may influence both adipose tissue function and insulin sensitivity (137). Interestingly, SPARC blood concentration has been found to increase in T2DM (138). Epidemiological research has also assessed the prognostic potential of myokines as biomarkers for bone health in diabetes. The median plasma DKK3 concentration is 32.8 ng/mL, with an interquartile range (IQR) of 28.0–39.0 ng/mL (139). Urinary DKK3 serves as a kidney-specific biomarker associated with tubular stress and fibrosis, whereas plasma DKK3 levels integrate signals from various tissues, reducing its specificity for chronic kidney disease (CKD) (101, 139). Findings suggest that baseline myokine levels can serve as predictors for future bone loss and fracture risk, underscoring their value as diagnostic and prognostic tools. Nonetheless, the variability in study methodologies, patient demographics, and myokine assay techniques across different studies calls for further validation through large-scale, well-controlled clinical trials. In summary, both animal models and human studies provide compelling evidence that myokines are integral to the pathophysiology of diabetes-related bone disorders. While current findings are encouraging, there is a pressing need for more extensive research to unravel the precise mechanisms through which myokines modulate bone health in the context of diabetes and to evaluate their potential as therapeutic targets.

## 5 Therapeutic strategies targeting myokines to enhance bone health in diabetic and patients

The recognition of myokines as essential regulators of muscle-bone crosstalk has opened new therapeutic avenues for addressing osteoporosis, particularly in diabetic patients. Given that diabetes can influence bone metabolism in complex ways through altered myokine expression, increased inflammation, and impaired bone remodeling, targeting myokines represents a promising strategy to mitigate osteoporosis and fracture risk in these populations. Multiple therapeutic approaches are currently being explored, including exercise interventions, myokine mimetics, and gene therapies.

### 5.1 Exercise interventions

Physical activity can stimulate the production of myokines (140, 141). Tailoring individualized exercise programs could also better modulate myokine expression and improve bone health. The specific effects of exercise-induced myokines on bone health in patients with T1DM and T2DM remain areas that require further research.

### 5.2 Myokine mimetics

Several myokine mimetics have entered clinical trials, offering promising therapeutic options for muscle-related disorders and osteoporosis. These mimetics aim to replicate the actions of naturally occurring myokines. Among the most studied GDF8 inhibitors in clinical research are MYO-029 (Anti-MSTN monoclonal antibody), Domagrozumab (Anti-MSTN monoclonal antibody), LY2495655 (Anti-MSTN monoclonal antibody), REGN1033 (Anti-MSTN monoclonal antibody), SRK-015 (Anti-propeptide monoclonal antibody), Bimagrumab (Anti-receptor monoclonal antibody), ACE-031 (Decoy receptor), and ACE-083 (Follistatin/Fc fusion protein). Broader inhibitors like Bimagrumab and ACE-031 have demonstrated significant effects on muscle mass. However, these inhibitors are also associated with risks of adverse effects, particularly those impacting the vascular system. The specificity of these inhibitors is crucial, as it influences their potential to cause off-target effects (142, 143). Particularly, Bimagrumab emerges as a significant therapeutic agent of interest. In the phase 2 clinical trial, it was demonstrated that Bimagrumab, an inhibitor of activin type II receptors (ActRII), led to a substantial reduction in body fat mass and concurrently enhanced glycemic control among adults afflicted with type 2 diabetes and obesity (144). Although clinical trials are underway, the effects of myokine mimetics on bone still require further investigation, particularly within the complex context of diabetes. More research is needed to fully understand the role of these mimetics in this condition.

### 5.3 Gene therapy

Direct delivery of myokine genes or gene encoding sequences to muscle tissue might lead to increased local levels and could in turn support bone health. This article focuses on two well-established myokine-based gene therapies: GDF8 and FGF21. The study explores AAV8-mediated delivery of GDF8 propeptide as a therapy in both normal and dystrophic (mdx) mice, demonstrating enhanced muscle growth and improved dystrophic symptoms. AAV8-mediated GDF8 inhibition is identified as a promising strategy for treating muscular dystrophy (145). Additionally, another study investigates the potential of fibroblast growth factor 21 (FGF21) gene therapy using AAV vectors to counteract obesity, insulin resistance, and T2D. Researchers showed that liver-specific AAV-mediated expression of FGF21 had significant and long-lasting effects in high-fat diet (HFD)-fed mice and genetically obese ob/ob mice, and also prevented age-associated metabolic decline in healthy animals (146). While there is ongoing debate about whether FGF21 negatively affects bone mineral density, the effects of these myokine-based gene therapies on bone health, particularly in the context of osteoporosis and diabetes, have yet to be fully studied. Therefore, when considering gene therapy for osteoporosis under physiological and diabetic conditions, it is essential to assess not only the effects of myokines on bone health but also their influence on whole-body metabolism.

## 6 Current limitations in understanding the role of myokines in bone metabolism in diabetes

### 6.1 Diversity of myokines and their targets

The range of myokines involved in bone metabolism is extensive. While most animal studies have focused on global myokine knockouts, many findings remain controversial and require further experimental validation. Current research, based on reproducibility of results, supports that GDF8 and GDF11 are well-established as negative regulators of bone metabolism, while Musclin, SPARC, and Irisin have been identified as positive regulators. Nonetheless, additional studies are necessary to confirm and refine these roles. Moreover, research specifically examining muscle-specific myokine knockouts and their effects on bone remains limited. In bone-specific investigations, critical factors such as mouse strain, age, sex, and diet composition must be carefully considered, yet many of these variables are underexplored or poorly understood, particularly in the context of diabetes. This highlights the need for more focused research in this area (**Table 2**). Furthermore, the full spectrum of muscle-derived factors influencing bone remodeling in diabetic conditions has not been fully characterized. The possibility remains that other yet-to-be-identified myokines may have significant effects on bone homeostasis and pathology in diabetes, emphasizing the importance of comprehensive analyses to further elucidate these complex relationships.

**Table 2:** Summary of reported levels of selected myokines in individuals with type 1 diabetes mellitus (T1DM) and type 2 diabetes mellitus (T2DM).

Changes in Myokine Levels in Type 1 and Type 2 Diabetes						
Myokines	Normal level	Ref.	T1DM	Ref.	T2DM	Ref.
Irisin (ng/mL)	3 – 5	Estell, 2020 [22]	↑	Espes, 2015 [117]	↓	Vecchiato, 2022 [118] Du, 2016 [119]
	~100	Huh, 2012 [44]				
GDF8 (ng/mL)	1 – 8	Meloux, 2019 [120] Zheng, 2022 [121]	↑	Dial, 2020 [122]	—	Brandt, 2012 [123]
GDF11 (ng/mL)	5.92 ± 2.52	Chen, 2016 [124]	Not reported		—	Añón-Hidalgo, 2019 [125]
IL-6 (pg/mL)	5.186	Said, 2021 [126]	↑	Jin, 2024 [127]	↓	Kristiansen, 2005 [128]
BDNF (ng/mL)	32.69 ± 8.33	Naegelin, 2018 [129]	↓	Chen, 2021 [130]	↑	Krabbe, 2007 [131]
FGF21 (pg/ml)	100–200	Taniguchi, 2022 [132]	↓	Taniguchi, 2022 [132]	↑	Keuper, 2020 [133] Geng, 2020 [134]
Musclin (ng/mL)	66.35 – 104.33	Zhang, 2023 [135]	Not reported		↑	Zhang, 2023 [135] Chen, 2017 [136]
SPARC (ng/mL)	11.72 ± 4.47	Kos, 2010 [137]	Not reported		↑	Wu, 2011 [138]
DKK3 (ng/mL)	32.8 (IQR: 28.0–39.0)	Piek, 2021 [139]	Not reported		Not reported	

Arrows indicate the direction of change (↑ increased; ↓ decreased; — no significant change). Where information was not available, it is marked as “Not reported.”

### 6.2 Complexity of diabetes and muscle-bone crosstalk

Diabetes presents a complex pathophysiological challenge, encompassing hyperglycemia, insulin resistance, inflammation, and the accumulation of advanced glycation end products (AGEs). The interplay between these factors and myokines, and their collective impact on bone metabolism, remains poorly understood. Our previous research has shown that bone health is significantly compromised in both T1DM and T2DM (10, 147), particularly due to disruptions in osteoblast glucose metabolism. However, we have observed that focusing solely on bone cell metabolism is insufficient to prevent long-term bone loss. A more comprehensive approach is necessary, one that addresses the broader pathophysiological context of diabetes, including the influence of muscle on bone health. This complexity is further amplified by the bidirectional communication between muscle and bone, which is disrupted under diabetic conditions. This makes it challenging to isolate the specific contributions of myokines from the wider metabolic disturbances associated with diabetes, underscoring the need for a more integrated understanding of these interactions.

### 6.3 Methodological challenges

One of the key challenges we have faced is the variability in detection methods for myokines, such as Irisin. Different methodologies produce inconsistent concentration measurements, which in turn lead to discrepancies in the experimental concentrations used. As a result, this variability has contributed to contradictory findings in our studies, underscoring the need for standardized detection approaches. Investigating the effects of myokines on bone metabolism *in vivo* is fraught with methodological difficulties. One major challenge is distinguishing the direct effects of myokines on bone cells from the myriad systemic changes occurring in a diabetic state, such as altered nutrient availability, hormonal imbalances, and inflammatory responses. This makes it difficult to attribute observed changes in bone health solely to myokine activity without the confounding effects of other factors.

## 7 Future research directions

### 7.1 Impact of diabetes on myokine production and function

Research should explore how diabetic conditions—characterized by hyperglycemia, insulin resistance, and systemic inflammation—affect the production and function of myokines. Systematically investigating the regulation of myokine expression and secretion under diabetic stress could provide insights into their roles in bone metabolism and highlight potential points for therapeutic intervention.

### 7.2 Identification of novel myokines

Future research should prioritize the identification and characterization of novel myokines that may influence bone metabolism, particularly under diabetic conditions. This could be achieved through comprehensive proteomic and transcriptomic analyses of muscle tissues from diabetic animal models and human patients, which could reveal new candidates for further investigation. Understanding which specific subpopulations of muscle cells are responsible for secreting myokines remains a pivotal area of investigation for future research. The advent of scRNA-seq technology provides an unparalleled opportunity to delve into this question with great specificity. By applying single-cell sequencing, researchers can distinguish between different muscle cell types, such as satellite cells, myoblasts, and myocytes, and determine their unique roles in myokine production. To further elucidate the function of specific myokines within the context of diabetes, muscle-specific Cre-loxP strategies can be employed to selectively knock out myokine genes in particular muscle cell subtypes. This targeted genetic approach allows researchers to validate the contribution and mechanistic role of these myokines in the pathophysiology of diabetes, including their effects on muscle-bone crosstalk, metabolic regulation, and systemic inflammation. By combining single-cell sequencing with muscle-specific gene editing, it becomes feasible to map out the cellular and molecular landscapes that drive myokine secretion and to better understand how these factors contribute to diabetic complications and overall muscle health.

### 7.3 Elucidation of myokine signaling pathways

There is a need for detailed studies focused on the specific signaling pathways activated by myokines in osteoblasts lineage cells and osteoclasts. Understanding these pathways could uncover new molecular targets for therapeutic intervention, helping to mitigate the adverse effects of diabetes on bone health.

### 7.4 Therapeutic potential of myokines

Given the emerging role of myokines in bone metabolism, research should continue to explore their therapeutic potential. Strategies could include developing drugs that enhance the production of beneficial myokines, mimic their effects, or inhibit the actions of detrimental myokines in diabetic patients with bone complications.

### 7.5 Clinical trials and marker development

Conducting clinical trials to validate the efficacy of myokine-based therapies in diabetic populations is essential. Additionally, the development of markers that reflect the activity of myokines could provide a valuable tool for monitoring treatment responses and predicting long-term outcomes in patients with diabetes-related bone disorders.

## 8 Conclusion

Myokines are emerging as critical regulators of bone health, particularly in the context of osteoporosis. They mediate the communication between muscle and bone, influencing bone remodeling and mass. As our understanding of these molecules deepens, myokines could become central to new strategies for managing osteoporosis and related disorders. Our review provides a comprehensive and detailed analysis of the bone phenotypes associated with each myokine, as discrepancies in the results may partially stem from these factors. This in-depth examination not only identifies potential sources of inconsistency but also offers valuable insights into the specific roles myokines play in bone physiology. Notably, even under physiological conditions, the bone phenotypes of certain myokines remain a topic of debate. Furthermore, research that isolates the effects of muscle-derived factors on bone is still limited, with the majority of studies relying on global knockout mouse models. In the context of diabetes, particularly T1DM, both animal and clinical studies remain underexplored, highlighting the need for further research in these areas. With the continued advancement of proteomics and single-cell omics, these technologies offer the potential to identify a broader range of myokines and precisely determine the specific cell types responsible for their secretion. Furthermore, they provide an opportunity to systematically elucidate the roles of myokines in bone metabolism within the context of T1DM and T2DM, offering deeper insights into their impact under diabetic conditions. The therapeutic targeting of myokines holds great potential for treating osteoporosis. By modulating the activity of specific myokines, it may be possible to enhance bone density, reduce fracture risk, and improve overall musculoskeletal health, offering a promising avenue for future research and clinical applications​.
